# Mechanically Induced Cavitation in Biological Systems

**DOI:** 10.3390/life11060546

**Published:** 2021-06-10

**Authors:** Chunghwan Kim, Won June Choi, Yisha Ng, Wonmo Kang

**Affiliations:** School for Engineering of Matter, Transport and Energy, Arizona State University, Tempe, AZ 85281, USA; ckim110@asu.edu (C.K.); wchoi37@asu.edu (W.J.C.); ywng1@asu.edu (Y.N.)

**Keywords:** cavitation, soft matter, blunt injury mechanism, dynamic bubble behaviors, acceleration-induced pressure gradients

## Abstract

Cavitation bubbles form in soft biological systems when subjected to a negative pressure above a critical threshold, and dynamically change their size and shape in a violent manner. The critical threshold and dynamic response of these bubbles are known to be sensitive to the mechanical characteristics of highly compliant biological systems. Several recent studies have demonstrated different biological implications of cavitation events in biological systems, from therapeutic drug delivery and microsurgery to blunt injury mechanisms. Due to the rapidly increasing relevance of cavitation in biological and biomedical communities, it is necessary to review the current state-of-the-art theoretical framework, experimental techniques, and research trends with an emphasis on cavitation behavior in biologically relevant systems (e.g., tissue simulant and organs). In this review, we first introduce several theoretical models that predict bubble response in different types of biological systems and discuss the use of each model with physical interpretations. Then, we review the experimental techniques that allow the characterization of cavitation in biologically relevant systems with in-depth discussions of their unique advantages and disadvantages. Finally, we highlight key biological studies and findings, through the direct use of live cells or organs, for each experimental approach.

## 1. Introduction

When a homogeneous liquid is subjected to a transient pressure drop below its saturated vapor pressure at a given temperature, small vapor cavities, referred to as *Cavitation* [1], can be formed inside the liquid media. Generally, cavitation can be classified into two types: *Inertial* and *Non-inertial*. The former describes rapid bubble dynamics that involve unstable bubble expansion and collapse typically triggered by a rapid change of pressure with a relatively large amplitude. The latter refers to much gentler bubble dynamics, e.g., the stable oscillation of a bubble around its equilibrium radius, typically driven by small periodic external pressure. Inertial cavitation dynamics, the focus of the current review, involves multiple steps including nucleation, expansion, oscillation, and collapse. During the bubble expansion, the bubble works against the resistance of the surrounding media, i.e., liquid. During the bubble collapse, the energy stored in the media is released. This collapse is violent in nature because the energy release is very localized at very high rates, a phenomenon known as *microjetting*.

Traditionally, cavitation in liquid has been of great interest to many researchers due to its important implications for many industrial and military applications. For example, sudden pressure drops in liquid media can occur in many engineering systems that involve rapid acceleration of the media, such as propellers of submarines and ships, hydraulic pumps, water turbines, and industrial piping systems. Due to its violent nature, cavitation can damage even the strongest man-made materials and structures over time, significantly shortening the life of these systems. Therefore, traditional research has focused on preventing cavitation-induced damage by predicting and avoiding the critical conditions that trigger cavitation nucleation.

There have been increasing research efforts to investigate cavitation in biological systems, e.g., a human body or tissue simulant. For example, significant progress has been made in shockwave lithotripsy (SWL) [2,3,4,5,6] by understanding the contribution of cavitation dynamics for biomedical applications [7,8,9,10,11]. Similarly, laser-induced cavitation has been used in ophthalmic microsurgery [12,13]. Another biomedical application of cavitation is targeted drug delivery [14,15,16]. In these works, cavitation was used to release an encapsulated drug within a carrier, such as a liposome or polymeric nanoparticle, when the carriers were near the target site, e.g., tumor or cancer. Other than that, a microfluidic system with highly controllable bubbles also gives us several advantages associated with understanding of cell injury mechanism or mechanotransduction via calcium signaling processes [17,18,19]. In the viewpoint of being possible for single-cell analysis, it is helpful to characterize shear stress-induced membrane deformation and the level of its poration.

More recently, several studies have reported that injuries that involve rapid acceleration of the human body by mechanical impact, e.g., car crash, collisions during sporting events, and bullet wounds [20,21,22,23], can induce cavitation in the human body or a tissue simulant. Among the instances of cavitation in the human body, cavitation-induced traumatic brain injury (TBI) has received much increased attention, because cavitation bubbles inside the human skull can result in tremendous brain damage [24,25,26]. Therefore, it is essential to understand the behavior of bubbles from the nucleation of the cavity to the collapse of the bubble and its effect on biological systems.

With biomedical applications of cavitation, there have been rapidly increasing demands for theoretical and experimental characterization of cavitation dynamics in biological systems to capture the unique interplay between cavitation and soft biological systems. Unlike homogeneous pure liquid, cavitation in soft biological systems exhibits highly complex behavior due to the viscoelastic properties [27,28,29] and heterogeneous microstructures [30,31] of biological systems. In this regard, we reviewed recent research progress on theoretical and experimental approaches for investigating cavitation dynamics in biological systems and biomedical applications. First, we introduce various strain energy function-based constitutive models that delineate bubble behavior in a wide range of biological matters. Each model is described with its physical implications. Then, we consider four different types of experimental methods—needle/acoustic/laser-induced cavitation and an integrated drop tower system—to investigate cavitation phenomenon in the scope of biological applications. Finally, we highlight key in vitro and in vivo studies.

## 2. Theoretical Background: Static and Dynamic Approaches

Following the seminal work by Rayleigh [32], it has been shown that the response of inertial cavitation bubbles in media (e.g., liquid or soft materials) depends on the material properties of the media, such as its surface tension [33], viscosity [34] and material stress tensor (σ) associated with the deformation of the media due to change in bubble size and shape. Two different theoretical approaches (i.e., static and dynamic) are available to analyze cavitation bubbles. These two approaches offer crucial theoretical frameworks for interpreting experimental observations from recently developed experimental techniques. It is worth noting that our emphasis is on the dynamic approach since detailed review on the static approach is available elsewhere [31,35,36,37,38,39].

### 2.1. Static Approach

The static approach is mostly used to predict the critical bubble size that corresponds with the onset of unstable bubble growth, known as *bubble burst*, without considering the time-dependent behavior of cavitation. This approach is applicable when bubble size changes very slowly and, therefore, dynamic effects can be ignored.

When a spherical cavitation bubble changes its size in a soft material sample, the stress tensor is developed due to the interplay between the bubble and soft material. Using a nonlinear Kelvin–Voight model [40,41], the tensor consists of elastic stress ($\sigma_{e}$) and viscous stress ($\sigma_{v}$) as follows:

(1) $$\sigma =\sigma_{e}+\sigma_{v}$$

$\sigma_{e}$ depends on the current deformation of the soft material sample and $\sigma_{v}$ is strain-rate-dependent (i.e., time-dependent). In the static approach (i.e., a bubble in a soft material sample deforms very slowly), the second term in Equation (1) (i.e., $\sigma_{v}$) is not considered.

When the soft material sample is hyperelastic, isotropic, and incompressible, the elastic stress tensor ($\sigma_{e}$) in the sample can be defined by a function of strain energy density (*W*) as follows [42]:

(2) $$\sigma_{e,ij}=2\left[\left(\frac{\partial W}{\partial I_{1}}+I_{1}\frac{\partial W}{\partial I_{2}}\right)V_{ij}-\frac{\partial W}{\partial I_{2}}V_{ik}V_{kj}\right]$$

where $I_{i}$ is an *i-*th invariant, *V* is the left Cauchy–Green strain tensor (*V* = *FF*^T^, where *F* is the deformation gradient tensor [42]), *i* and *j* are free indices, and *k* is a dummy index.

To predict the behavior of cavitation bubbles in different types of soft materials, several constitutive models have been developed and utilized for the invariant of the Cauchy–Green strain tensor (see Table 1). For the neo-Hookean (NH) model [43], the strain energy density is expressed only by the first invariant ($I_{1}$, i.e., hydrostatic stress) of the tensor V. In addition to $I_{1}$, the Mooney–Rivlin (MR) model [44,45] includes the second invariant ($I_{2}$, i.e., distortional stress), where the strain energy of isotropic material is a symmetric function of $I_{1},\;I_{2}\;\mathrm{and}\;I_{3}$ where $I_{3}=1$. By including the second invariant, the MR model provides a wider range of responses of hyperelastic material compared to NH [46], as it considers the deformation of a soft gel by both the mean normal stress tensor (i.e., $I_{1}$) and the deviatoric component (i.e., the stress deviator tensor or $I_{2}$). Gent [47] developed a new model that defines the maximum value of $I_{1}$ (referred to as $I_{m}$) in the NH model. $I_{m}$ is introduced to describe the state of polymer chains in a hydrogel. As $I_{1}$ approaches $I_{m}$, the entangled polymer chains are straightened, aligned, and axially stretched, which results in rapid stiffening. Another model is the Ogden model [48,49], which consists of polynomial terms that capture the material deformation in the principal directions (see Table 1 for more details). It has been experimentally shown that the Ogden model captures cavitation dynamics in gelatin gels, commonly used as tissue simulant, as well as in different types of organs [46,50,51]. Fung [52,53] developed a constitutive model that takes the strain hardening effect [54] into account, e.g., the effect of pre-stretched soft tissues on elastic shear measurements [54]. Table 1 summarizes the mathematical expressions of the constitutive models discussed here.

When a soft gel is incompressible, the principal stretch of a cavitation bubble due to applied pressure, p, can be described in the spherically symmetric coordinate as follows [55,56]:

(3) $$p=p_{o}+\frac{2\gamma }{a}+\int_{1}^{\lambda }\frac{W`\left(\lambda \right)}{\left(\lambda^{3}-1\right)}d\lambda$$

where $p_{o}$ is the ambient pressure, a is the deformed bubble radius, γ is the surface tension of gel, and λ is the normalized radius of the cavitation bubble. Note that a different constitutive model can be substituted into Equation (3). The applied pressure p is balanced with the ambient pressure, the Laplace pressure due to surface tension, and the stress term associated with the deformation of gel [55]. it is worth noting that there have been recent studies that consider additional effects from pH [57], temperature [58], nonlinear elasticity [59], humidity [60,61], and energy dissipation level [62], which is beyond the scope of this review. 

### 2.2. Dynamics

Here, the focus is placed on the time-dependent behavior of a spherical cavitation bubble in soft material (i.e., $\sigma_{v}$ in Equation (1)). The viscous stress $\sigma_{v}$ is a deviatoric and linearly dependent on a strain rate as follows [41]:

(4) $$\sigma_{v}=\nu \left(\nabla u+\nabla u^{T}\right)$$

where u=dr/dt, *t* is time, and ν is the viscosity coefficient of the soft material. Substituting u(r,t) and σ into the radial component of the momentum equation, the governing equation of a spherical cavitation bubble in soft material can be written as follows [63]:

(5) $$\rho \left(\frac{\partial u}{\partial t}+u\frac{\partial u}{\partial r}\right)=-\frac{\partial p}{\partial r}+{\left(\nabla \cdot \sigma \right)}_{r}=-\frac{\partial p}{\partial r}+\frac{\partial \sigma_{rr}}{\partial r}+\frac{2\sigma_{rr}-\sigma_{\theta \theta }-\sigma_{\phi \phi }}{r}$$

where *r* is the radial coordinate from the center of the bubble at the deformed state, ρ is density, θ, and ϕ are the polar and azimuthal angle in the spherical coordinate configuration. $\sigma_{rr}$, $\sigma_{\theta \theta }$, and $\sigma_{\phi \phi }$ are the components of the Cauchy stress tensor in the spherical-polar representation. Integrating the above Equation (5) with the stress tensor given in Equation (1) results in

(6) $$a\ddot{a}+\frac{3}{2}{\dot{a}}^{2}=\frac{p\left(a\right)-p_{\infty }\left(t\right)}{\rho }+\frac{1}{\rho }\int_{a}^{\infty }{\left(\nabla \cdot \sigma \right)}_{r}dr$$

where $p_{\infty }\left(t\right)$ is the pressure in the medium far from the bubble and p(a) is the pressure in the medium at the bubble-medium interface. Note that the integration in Equation (6) is evaluated over an infinitely large medium, i.e., from the current bubble radius (r=a) to infinity (r=∞). Finally, the following governing equation can be obtained from Equation (6):

(7) $$\frac{p_{B}-p_{\infty }\left(t\right)}{\rho }=a\ddot{a}+\frac{3}{2}{\dot{a}}^{2}+\frac{2\gamma }{\rho a}+\frac{4\mu \dot{a}}{\rho a}+\frac{1}{\rho }\left(\int_{1}^{\lambda }\frac{W`\left(\lambda \right)}{\left(\lambda^{3}-1\right)}d\lambda \right)$$

where $p_{B}$ is the internal bubble pressure and μ is the viscosity of the medium, and the over dot indicates the derivative of a with respect to time. When $p_{B}$ is a polytropic process, it can be expressed as $p_{B}=p_{v}+\left(p_{\infty }\left(t=0\right)+2\gamma /A-p_{v}\right){\left(\lambda \right)}^{3k}$, where $\;p_{v}$ is the vapor pressure, *k* is the ratio of the specific heat, i.e., the polytropic index. Equation (7) is the Rayleigh–Plesset (RP) equation, where the last term considers the effect of soft material deformation on the bubble dynamics. As discussed above, a different constitutive material model can be utilized to analyze different biological soft materials. For example, the RP equation with the neo-Hookean (NH) model has been widely utilized to analyze experimentally measured cavitation bubble behaviors in soft hydrogels [10,27,28,29,64,65].

So far, we have introduced the governing equation of single bubble dynamics in the Kelvin–Voigt-type constitutive model, represented by a viscous damper and an elastic spring in parallel, that captures the creep behavior of soft media [40]. Here we discuss other available linear viscoelastic models: the linear Maxwell and solid models. The Maxwell model, composed of a purely elastic spring and a purely viscous damper in series, is applicable for liquid-dominant materials [40] (see Table 2). The linear solid model, a combination of the Kelvin–Voigt and Maxwell models, is used to describe creep, deformation recovery, and stress relation in soft media.

It is worth noting that there have also been continuous research efforts to modify the RP equation to include mass and thermal transfer, compressibility [73,74], non-spherical perturbations [75], and larger deformation of soft materials, e.g., by developing more complex nonlinear constitutive models [76,77,78,79]. The details are not discussed as these topics are beyond the scope of the current review.

## 3. Experimental Methods for Cavitation-Induced Damage to the Biological Systems

Here, we review experimental techniques for triggering and analyzing cavitation bubbles in biologically relevant systems. We categorize the available experimental techniques into two groups: static (needle-induced cavitation) and dynamic (laser- and acoustic-induced cavitation and the integrated drop tower system). We directly compare the advantages and disadvantages of these newly developed techniques with an emphasis on cavitation-induced damage to biological systems (i.e., mechanisms of blunt injuries). A few key biological advances utilizing each technique are also highlighted.

### 3.1. Needle-Induced Cavitation

**Figure 1:**
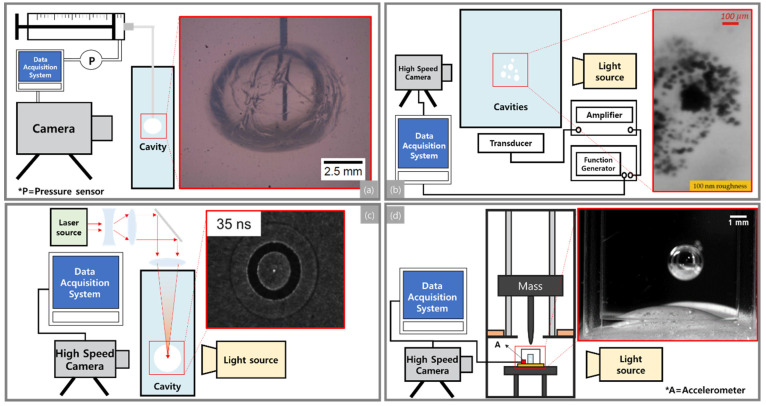
Schematic diagrams and cavitation images of (**a**) NIC (Needle-induced cavitation), (**b**) AIC (Acoustically induced cavitation), (**c**) LIC (Laser-induced cavitation), and (**d**) integrated drop tower system. Regenerated by permission from the following references [28,31,36,80,81,82,83].

Needle-induced cavitation (NIC), shown in Figure 1a [31,36], was developed by Crosby’s research group [31]. A cavitation bubble in a soft material sample was created by applying pressure through a narrow needle inserted into the sample. The needle was connected to a syringe and a pressure sensor by small tubes so that the applied pressure was precisely controlled by concurrently utilizing a syringe pump and pressure sensor. Bubble size was monitored by using a microscope as applied pressure incrementally increased.

The NIC method allows experimental characterization, which correlates the mechanical properties of soft material samples (e.g., elastic modulus and viscosity) with their critical pressure at the onset of the bubble burst [31,37]. To improve the accuracy of the characterization, more detailed studies have followed, including the study of cavitation behavior (cavitation and/or fracture) resulting from differing needle diameters ranging from 30 to 205 μm and differing polymer compositions [35,36,39].

**Figure 2:**
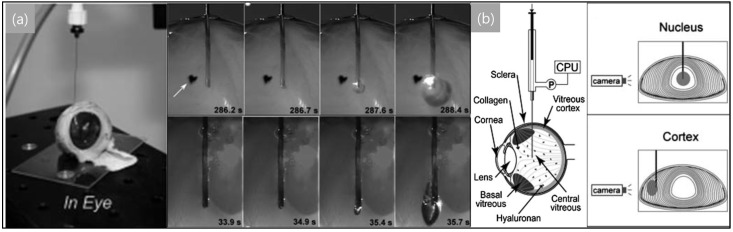
Images (**a**) and schematic diagrams (**b**) of the needle-induced cavitation method (NIC) introduced by Crosby’s research group. Using the NIC method, cavitation behavior and mechanical properties of the bovine eye were investigated [84,85].

Due to its simple working mechanism, the applications of the NIC method have been expanded to biological organs, e.g., eyes, skin, and bone marrow, at relatively low strain rates (10^−1^–10^−3^ s^−1^). Zimberlin et al. [31] first demonstrated the use of NIC for in vivo samples [84] by measuring the elastic modulus of the bovine eye (more specifically, the vitreous body in an eye (shown in Figure 2) [84,85]. Similarly, it has been reported that biological organs have location-dependent elastic moduli (e.g., the elastic moduli measured in the areas of the nucleus and cortex parts in an extracted bovine eye (see Figure 2) were 11.8 and 0.8 kPa, respectively [85]).

NIC-based methods, unlike conventional shear rheometry for bulk elastic modulus, allow the measurement of localized elastic modulus in heterogeneous soft material samples. In addition, the method is relatively simple and cheap [31]. Furthermore, the size of a void can be controlled by the needle radius, gas pressure, and pressure rate. Despite these advantages, it is difficult to use the NIC method when the length scale of the defects is in the same order as the cavitation size. In addition, the NIC method is mainly for quasi-elastic behavior due to its slow strain rate (about 10^−1^–10^−3^ s^−1^).

### 3.2. Acoustically Induced Cavitation

Acoustically induced cavitation (AIC) uses ultrasound as the driving force of cavitation nucleation and oscillation. Typically, the AIC system (see a schematic in Figure 1b [82,83]) consists of transducers, signal amplifiers, and waveform generators for generating and controlling desired ultrasonic inputs to biological samples. When a liquid is subjected to an ultrasound field, alternating expansion and compression cycles occur in the media. If the intensity of the alternations is sufficiently large, pressure decreases rapidly and gaseous bubbles nucleate [86].

It is important to note that the AIC method utilizes acoustic fields in soft materials and, as a result, it typically nucleates many cavitation bubbles. Because of this feature, it is not trivial to use the AIC method to characterize material properties of soft materials. To overcome this experimental challenge, Mancia et al. proposed a cavitation rheometry technique that uses highly focused ultrasound to generate a bubble, named for inertial microcavitation-based high strain rate rheometry (IMR), which has the high strain rate range from 10^3^ to 10^8^ s^−1^. Using this new method, the mechanical properties of agarose hydrogel were quantified [87].

The AIC method has garnered significant attention especially in biological systems due to its ability to focus energy on a small volume. One of the early uses of ultrasound in biological applications was reported by Brohult et al. to study the degradation of biological polymers [88]. This pioneering work gave rise to increasing efforts to characterize how ultrasound interacts with biological systems in the scope of establishing the criteria for safe use of ultrasound in medical applications. For example, Pohlman et al. investigated the diminishing intensity of ultrasound beams when transmitted through several layers of tissue [89]. Carstensen and Schwan et al. focused on the reduced intensity of ultrasound waves as they propagated through blood [90]. Owing to this prior work, the AIC method has been used for many biomedical applications such as disintegration of kidney and gallstones (shockwave lithotripsy) [5,91,92,93,94] and intracellular delivery of molecules to a target site (drug delivery) [95].

In shockwave lithotripsy (SWL), several studies revealed the importance of cavitation collapse for in vitro applications [9,96,97,98,99,100,101]. Ikeda et al. proposed high-intensity focused ultrasound (HIFU) for lithotripsy to maximize the cavitation effect using two acoustic waves with different frequencies: one to nucleate multiple bubbles, called bubble clouds, and the other one to excite the bubble dynamics [8]. HIFU also showed great potential as a noninvasive treatment as SWL with the accurate control of cavitation behavior [11,102,103,104]. It is worth noting that excessive energy generated by SWL may result in considerable damage to organs, e.g., rupture of injury-prone blood vessels [105]. For example, it has been shown that bubble growth and collapse can lead to vessel stretching and vascular rupture [106,107,108]. To reveal these injury mechanisms, Chen et al. developed an experimental setup (Applied pressure: 4–7 MPa) that consists of a high-speed camera and an inverted microscope for spatial–temporal observations of cavitation bubbles near blood vessels [109].

**Figure 3:**
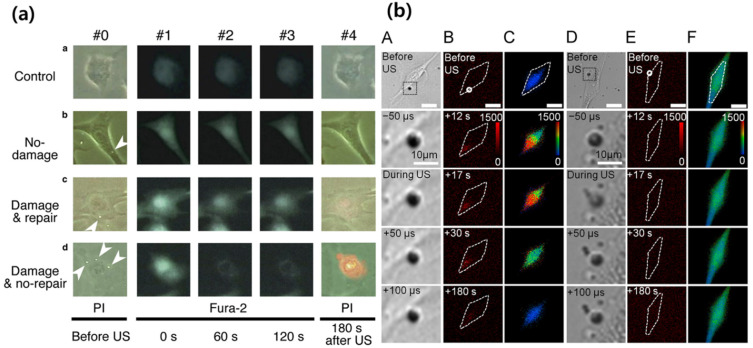
Cell membrane damage in the presence of a microbubble oscillated by ultrasound. (**a**) Damage and repair of bovine endothelial monolayer cells measured over time in propidium iodide (PI) and Fura 2 fluorescence [111]. (**b**) Time-lapse results of PI (B,E) and Calcium (C,F) changes in bEnd.3 cells with (A,B,C) or without shear stress (D,E,F) [112].

For in vitro demonstrations of targeted drug delivery, it has been shown that microbubbles driven by ultrasound influenced the membrane permeability of live cells [110], perforation for endothelial cells [111], and the shear stress on cell walls [112], as shown in Figure 3. Other studies also showed that ultrasound is an effective way to transfer therapeutic agents to rats’ hearts [113] and the epidermal growth factor receptor (EGFR)-direct small inhibitory RNA to target cells for slowing tumor growth [114].

One notable advantage of the AIC method is that it can be utilized in noninvasive medical applications by controlling the frequency and amplitude of input acoustic waves from medical imaging to lithotripsy. Despite these applications, multifaceted bioeffects [115,116,117] and the fundamental root of in vivo cavitation are still not well understood, even with low-intensity ultrasound. The foremost reason is that the generation of a single cavitation bubble using AIC is quite challenging as it requires highly focused acoustic waves and precisely controlled wave frequency, amplitude, and damping. The analysis of bubble dynamics in biological samples is rather complex due to continuous bubble-to-bubble interactions. 

In response to the challenges above, a theoretical model (e.g., Bilayer Sonophore (BLS)) has been developed. The model underscores the capability of transferring oscillating ultrasound waves to the expanded or contracted intramembrane compartment [118]. In addition, Iida et al. measured bubble size and distribution using a laser diffraction method and compared their experimental results with computational predictions [119]. Furthermore, the size and lifetime of bubbles has been investigated to reveal the behavior of clustered bubbles in bubble clouds [120,121,122,123].

### 3.3. Laser-Induced Cavitation

Since the development of light amplification by stimulated emission of radiation (i.e., laser) [124,125], there have been many attempts to apply laser techniques in biological applications. The measurement method using lasers, so-called the laser-induced cavitation (LIC) method, was introduced by several researchers as early as the 1970s [126].

The focused laser beam (see Figure 1c) [80,81]) transmits energy to a specific area within a sample. When the temperature increases above the critical threshold temperature, cavitation bubbles form in the soft sample. Then, the cavitation bubbles are monitored through a high-speed camera.

Recently, the LIC method has been applied to the characterization of the dynamic response of soft material at high strain rates (10^1^–10^8^) with an emphasis on underlying injury mechanisms in the human body including for traumatic brain injuries [27]. Because the LIC is based on the focused laser beam, it can be used to probe the dynamics of cavitation bubbles at different locations within a sample. This is an attractive feature for characterizing localized material properties of soft gels. For example, experimentally measured bubble dynamics over time have been analyzed and compared with theoretical analysis to predict material properties at 10^3^–10^8^ strain rates [27]. Brujan et al. investigated the interaction of a single bubble with hydrogel and showed the relationship between the elastic modulus and bubble dynamics such as jetting behavior, jet velocity, bubble oscillation time, bubble migration, and bubble erosion. For example, polyacrylamide gel with 0.25 MPa elastic modulus has a maximum liquid jet velocity of 960 ms^−1^, which can infiltrate the elastic boundary thickness [127]. This jetting ejection and the tensile stress from the bubble collapse can influence the ablation process during short-pulsed laser surgery.

Laser-induced cavitation has been widely applicable as a useful tool for probing the physics of ablation in soft tissues [128,129,130], microsurgery in vivo [131], medical diagnostic [132], cell lysis [133], etc. Short-pulsed lasers such as holmium and erbium have been particularly studied since they have high absorption coefficients in water and pass fairly well through a low concentration of hydroxide quartz fibers [128]. Some studies focus on cavitation dynamics during pulsed laser ablation. Asshauer et al. focused on acoustic transients after bubble collapse since this rapidly changing pressure might inflict direct or indirect damage on adjacent tissues [129]. Several studies [131,132,133] have been conducted to determine potential uses of LIC for therapeutic purposes. The findings of these studies are: (i) the critical cavitation formation values are lower in vivo and bubble growth can be restricted by the biological matrix [131], (ii) the onset of cavitation occurs below the medical safety range when gold nanoparticles are used as a seed for lowering the cavitation threshold, conducive to reducing the thermal effect to surrounding tissue based on their in vitro study [132], and (iii) cavitation bubble growth was one of the main reasons for cell lysis such that the extent of growth was characterized with respect to the pulse energy and cellular surface density [133].

Advantages of the LIC method include its noncontact process, highly focused localization, and use of electromagnetic radiation with uniform wavelength, phase, and polarization. The focused energy of the LIC method formed bubbles with higher pressure compared to other methods [38] (see in Table 3). However, the LIC method is also limited due to the thermal effect during bubble generation, which can cause permanent damage in biological systems [134]. Additionally, dielectric breakdown of the surrounding material can render it unstable, resulting in uncertain shifts of intrinsic properties in confined areas [135,136].

**Figure 4:**
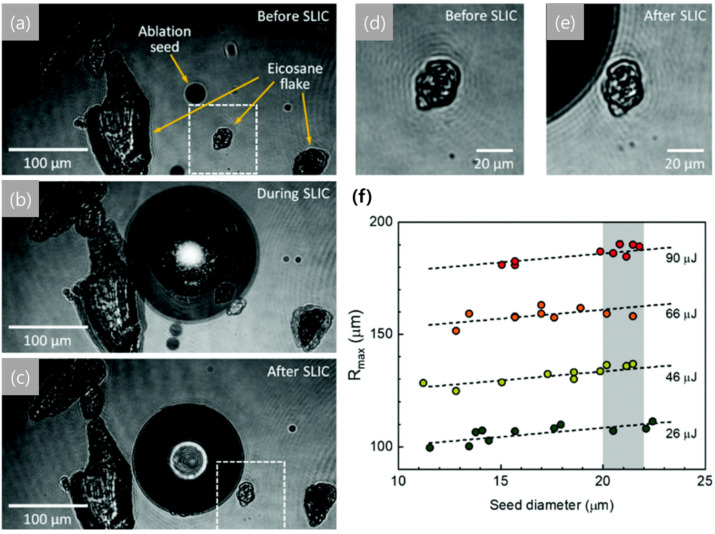
(**a**–**c**) Procedure of seeded laser-induced cavitation (SLIC) from cavitation nucleation to collapse. Magnified image of eicosane flakes around an ablation seed (**d**) before and (**e**) after SLIC. (**f**) Maximum cavity size with respect to seed diameter and laser-pulse energy [81].

Recent efforts to address the mentioned disadvantages are the following. Quinto-Su et al. examined the thermal distribution of the bubble after collapse using a high-speed camera [134]. There are several attempts to differ the laser source, i.e., using laser wavelengths from the near-infrared range, for example Nd:YAG source laser (1064 nm) to ArF excimer laser (193 nm), depending on the absorbance of materials and applications [137]. In addition, double- or multiple-pulse LIC methods, which use two or more laser pulses simultaneously, have been used recently to resolve limits of detection [138,139] and improve emission signal [140,141,142,143]. By minimizing the unpredictable inhomogeneous local material properties, Tiwari et al. made the best use of the geometrical flexibility of seeded laser-induced cavitation (SLIC), uncovering physical and dynamic bubble-to-tissue interactions over temporal and positional resolutions without disrupting any surroundings. As shown in Figure 4 [81], cavitation occurred at the aimed ablation seed, and the cavity expanded according to the increase in time. In this research, it was demonstrated that the SLIC method is an effective way to quantify the mechanical properties of soft matter, and in addition, when considering the shape and movement of seed before and after SLIC in Figure 4, the effect of the laser on temperature was not significant due to the low thermal diffusivity of the specimen and short time of laser application [81].

### 3.4. Integrated Drop Tower System

Characterizing and understanding cell and tissue response to rapid mechanical impact are crucial to the accurate assessment of potential blunt injuries and elucidating underlying injury mechanisms. When a human body is exposed to mechanical impact, the human skin, brain, or liver is rapidly accelerated, potentially resulting in acceleration-induced cavitation bubbles. As a result, mimicking the mechanical signatures of blunt injuries becomes essential for quantitative characterization of cell response under rapid pressure changes and cavitation events.

A recently developed experimental approach, called the drop-tower-based integrated system, allows the probing of the transient dynamic response of soft tissue simulant and live cells under well-controlled mechanical inputs. The drop-tower-based method (Figure 1d) consists of a unique sample holder and a series of effective springs and dampers which mimic common blunt injury events [144]. A known weight is lifted to a specific drop height and then released to apply impact to a sample. Each impact results in acceleration-induced pressure gradients in the sample. The response of the sample is recorded with a high-speed camera. This innovative method has been utilized to explore the effect of initial bubble size, shear modulus, and surface tension on cavitation bubble dynamics [28,29,144].

For example, Kang et al. have experimentally shown that impact-induced pressure gradients (100–400 kPa) in soft gels are sensitive to the size of the sample (proportional to the sample height squared). Furthermore, the critical transition in the material response from small deformations to sudden bubble bursts, also known as cavitation nucleation to growth, depends on the gel’s stiffness (3–200 kPa) as well as the initial size and shape of the bubbles. The key biologically relevant conclusions are (1) the establishment of the critical bounds of mechanical inputs which will likely result in cavitation-induced damage to biological systems; (2) that the size of biological systems, e.g., head size, should be appropriately considered for accurate assessment of potential injuries, because acceleration-induced local pressure strongly depends on the size of the sample. Fu et al. adopted the drop-tower-based method and introduced a microbubble into a sample by utilizing a microfluidic system [145]. This new approach allows control of the initial bubble size and its effect on bubble dynamics during mechanical impact.

**Figure 5:**
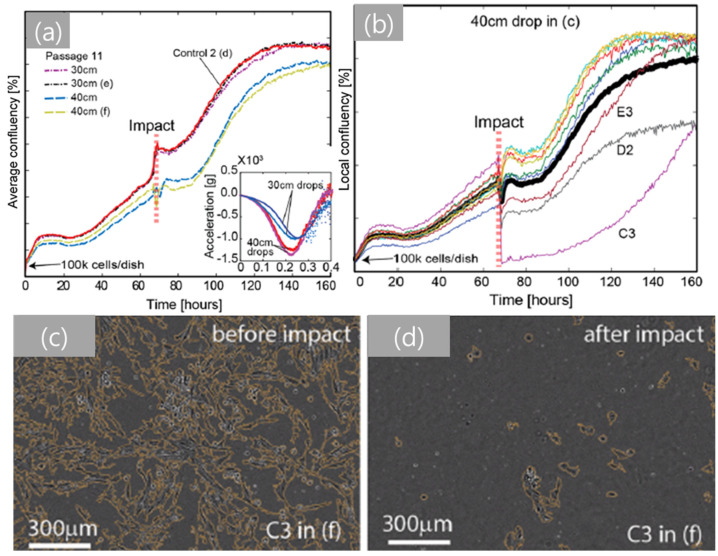
Characterization of cell injury depending on drop height using Hs27 fibroblasts (*x*-axis: time and *y*-axis: average confluency). (**a**) The average confluency graph for 30 and 40 cm drops. (**b**) The local confluency graph for 40 cm drop. (**c**,**d**) Live cell images during 40 cm drop (**c**) before and (**d**) after impact [146].

The drop-tower-based system has been recently modified for in vitro studies of live cells [146]. The innovative experimental setup allows the characterization of the experimental correlations between mechanical impact and cell damage/injury. This study, using fibroblast cells as a model, showed that input acceleration alone does not result in cell damage, as shown by Figure 5. However, cell membrane damage and a sudden decrease in cell population were observed above a material-dependent critical pressure value. These results indicate that the critical pressure is associated with the onset of cavitation bubbles in a cell culture chamber and that the dynamics of cavitation bubbles in the chamber induces localized compressive pressure cycles that significantly damage cells. This innovative technique could lead to new scientific findings on impact-induced cellular pathways that may trigger uncontrolled cell death (e.g., necrosis and apoptosis). Such findings would be an important step towards innovative technical advances in designing effective protective equipment and new biomedical technology for post-injury treatment of Service members.

In contrast to other methods such as AIC and LIC, the drop-tower-based system can correlate a physical or mechanical property, i.e., acceleration or its gradients, to cavitation nucleation in different types of soft matters. This capability to directly correlate the onset of cavitation with acceleration would be quite helpful to understanding the underlying injury mechanism of biomaterials that are known to be sensitive to strain rates. The drop weight impact test is currently the only method that allows control of the input acceleration profile. This unique feature is crucial to revealing blunt injury mechanisms as one can mimic exact acceleration profiles for actual blunt injuries and study the biological responses of live cells or tissues. Furthermore, this integrated system is coupled with a sample holder and high-speed cameras to avoid direct contact between the biological sample and the impactor while optically observing the real-time material deformation of soft gels. Despite the key findings of cellular damage at the population level correlated with changes in transient acceleration and the following bubble growth [146], there are still remaining questions, i.e., how this system can be used to analyze deformation and damage of individual cells during impact loading. Therefore, an effort to add high-resolution real-time imaging techniques, i.e., single-cell-level observations, to the current drop-tower-based system for in vitro studies would be critical to gain a fundamental understanding of the cavitation damage mechanisms at the single-cell/subcellular level.

## 4. Conclusions

The characterization, analysis, and interpretation of cavitation within biological matter are becoming inevitably important since they are increasingly relevant to medical applications such as lithotripsy, microsurgery, and medical imaging as well as to understanding blunt injury mechanisms. Due to emerging interests, there has been rapid technical advancement in the field of cavitation in biological systems. In this regard, we have reviewed cavitation in soft materials with an emphasis on biological implications of cavitation. First, the two main theoretical frameworks (static and dynamic approaches) have been discussed. Second, the experimental methods, i.e., needle-, laser-, and acoustically induced cavitation and the integrated drop tower system, have been discussed and directly compared with each other for different use cases and evaluated for their unique advantages and limitations.

## Figures and Tables

**Table 1:** The equation of elastic stress tensor and constitutive strain functions with different types of models with relevant material parameter.

Name of Model	Strain Energy Density (W)	Reference
Neo-Hookean Model	$\frac{\mu }{2}\left(I_{1}-3\right)$	[43]
Mooney–Rivlin Model	$\frac{\mu }{2}\left[c\left(I_{1}-3\right)+\left(1-c\right)\left(I_{2}-3\right)\right]$	[44,45]
Gent Model	$\frac{\mu }{2}I_{m}\ln\left(\frac{I_{m}}{I_{m}-I_{1}+3}\right)$	[47]
Ogden Model	$\frac{2\mu }{N^{2}}\left(\lambda_{r}^{N}+\lambda_{\theta }^{N}+\lambda_{\phi }^{N}-3\right)$	[49]
Fung Model	$\frac{\mu }{2\alpha }e^{\alpha \left(I_{1}-3\right)}$	[52,53]

**Table 2:** Summary of linear constitutive models [66].

Name of Model	Strain Energy Density (W)	Description	Reference
Newtonian	$\sigma_{rr}=2\nu {\dot{\varepsilon }}_{rr}$	Viscous stresses linearly dependent on the local strain rate	[67]
Kelvin–Voigt	$\sigma_{rr}=2\left(\mu \varepsilon_{rr}+\nu {\dot{\varepsilon }}_{rr}\right)$	A spring and a dashpot in parallel; Viscoelastic solid; Creep behavior	[41,68,69]
Maxwell	$2\nu {\dot{\varepsilon }}_{rr}=\frac{\nu }{\mu }{\dot{\sigma }}_{rr}+\sigma_{rr}$	A spring and a dashpot in series; Viscoelastic liquid; Stress relaxation	[70,71]
Standard Linear solid	$\frac{v}{\mu }{\dot{\sigma }}_{rr}+\sigma_{rr}=2\mu \varepsilon_{rr}+2\nu {\dot{\varepsilon }}_{rr}$	Both creep and stress relaxation	[72]

**Table 3:** Comparison of characteristics of cavitation methods (NIC, AIC, LIC, and drop-tower test) [38]. This table is modified from [38] with additional information.

	NIC	AIC	LIC	Drop Tower Test
Driving force	Pressure energy	Wave energy		Potential energy
Strain rate	10^−4^–10^3^	10^3^–10^8^	10^1^–10^8^	10^0^–10^5^
Scale (µm)	10^0^–10^5^	10^3^	10^−1^–10^2^	10^2^–10^5^
Pressure (Pa)	≤10^5^	≤10^7^	≤10^8^	≤10^6^
Cavity type	Single	Multiple	Single and Multiple	Single and Multiple
Level of accessibility	Low	High	High	Intermediate
Approach type	Contact	Noncontact	Noncontact	Noncontact
Thermal effect	Low	Intermediate	High	Low
Application	Drug delivery	Lithotripsy Imaging, Drug delivery	Drug delivery, Microsurgery, Medical diagnostic, High strain rate material properties	High strain rate material properties (Isothermal)

## Data Availability

3rd Party Data.
